# Modifiable life style factors and male reproductive health: a cross-sectional study in IVF clinic attendees in Ghana

**DOI:** 10.3389/frph.2025.1520938

**Published:** 2025-02-11

**Authors:** Brodrick Yeboah Amoah, Saliah Yao Bayamina, Cosmos Gborsong, Hubert Owusu, George Awuku Asare, Emmanuel Kwabena Yeboah, Josephine Ablakwa, Georgina Hammond

**Affiliations:** ^1^ Department of Medical Laboratory Sciences, School of Biomedical and Allied Health Sciences, University of Ghana, Accra, Ghana; ^2^ Department of Molecular Medicine, School of Medical Sciences, Kwame Nkrumah University of Science and Technology, Kumasi, Ghana; ^3^ Department of Medical Sciences, Royal Melbourne Institute of Technology, Melbourne, VIC, Australia; ^4^ Department of Anatomy and Physiology, Institute for Cardiovascular Prevention, IPEK, University of Munich, Munich, Germany

**Keywords:** semen quality, lifestyle factors, male infertility, reproductive hormones, IVF, Ghana, sperm motility, Ghanaian men

## Abstract

**Background:**

Male infertility is a significant global public health issue, with modifiable lifestyle factors such as smoking, obesity, and psychological stress contributing to impaired semen quality and hormonal dysregulation. This study investigates the relationships between modifiable lifestyle factors, reproductive hormones, and semen quality in Ghanaian males attending an IVF clinic.

**Methods:**

A cross-sectional study was conducted with 212 male participants recruited from a fertility clinic in Ghana. Lifestyle factors were assessed using standardized questionnaires, and semen samples were analyzed following WHO guidelines. Hormonal profiles (LH, FSH, testosterone, estradiol) were measured using the enzyme-linked fluorescent assay (ELFA). Statistical analyses included Pearson's product-moment correlation and Bonferroni correction.

**Results:**

Smoking and psychological stress were significantly associated with reduced sperm motility, viability, and concentration (*p* < 0.05). Elevated BMI correlated negatively with sperm concentration and testosterone levels (*p* < 0.05). Alcoholic bitters was linked to decreased semen quality, while caffeine consumption showed a positive association with progressive sperm motility.

**Conclusion:**

Modifiable lifestyle factors, such as smoking, psychological stress, and increased body mass index (BMI), play a crucial role in male reproductive health by adversely affecting semen parameters and hormonal balance. These findings emphasize the need for public health interventions targeting modifiable behaviors to improve fertility outcomes.

## Introduction

Infertility affects approximately one in six individuals globally, with male infertility accounting for nearly half of all cases (1). Male infertility is mostly linked to abnormal semen parameters and hormonal dysregulation, and is exacerbated by modifiable lifestyle and environmental factors (2). Clinically, infertility can be defined as the inability to become pregnant after 12 months or more of regular, unprotected sexual intercourse, manifesting as a failure to procreate (1). The condition is associated with significant psychological, socio-economic, and medical consequences, often leading to emotional distress, due to the inability to conceive.

**Table 1:** Lifestyle and environmental factors correlated with semen parameters.

Lifestyle Factors	Volume (ml)	Progressive motility (%)	Sperm viability (%)	Concentration (×10⁶/ml)
Alcohol consumption	0.313^a^	−0.087	−0.166^b^	−0.149^b^
Caffeinated drinks	−0.295^a^	0.399^a^	0.207^a^	0.223^a^
Smoking	0.136^b^	−0.317^a^	−0.450^a^	−0.084

^a^ Correlation is significant at the *p* < 0.01 level (2-tailed). ^b^Correlation is significant at the 0.05 level (2-tailed). Cor. Coeff. r, correlation coefficient; PR, progressive motility; NP, non progressive motility; Conc., sperm concentration.

Globally, infertility affects nearly 15% of couples, representing an estimated 186 million individuals, with male infertility contributing to more than half of these cases (2, 3). Notably, sperm counts in African males decreased by approximately 73% between 1965 and 2015 (4). Reports from Africa, including Nigeria, showed an increase in male infertility cases (5, 6).

Despite its significance, male reproductive health remains understudied and underfunded, with limited diagnostic and treatment facilities, especially in low-income settings. This neglect is exacerbated by the lack of male reproductive health policies, low-cost treatment options, and insurance coverage. Declining male fertility rates are linked to population decline, a critical public health concern for current and future generations (7).

Emerging evidence highlights the role of modifiable lifestyle factors such as smoking, alcohol consumption, and psychological stress in contributing to male infertility (8). These factors do not only impair sperm parameters but also induce DNA damage, increasing the risk of miscarriage and genetic disorders in offspring (9). Endocrine- disrupting chemicals (EDC), commonly found in pollutants, disrupt hormonal regulation and adversely affect spermatogenesis (8). Some studies have shown that these chemicals contribute to oxidative stress, which compromises sperm, and DNA integrity. For example, exposure to bisphenol A (BPA) and phthalates has been linked to reduced sperm motility and concentration (10). Additionally, psychological stress influences semen quality by altering hypothalamic-pituitary-gonal (HPG) axis function, further exacerbating infertility risks (11).

While moderate physical activity has been associated with improved semen quality, excessive exercise can negatively impact hormonal regulation and sperm parameters (12). Environmental pollutants, including heat exposure and radiation, also play a pivotal role in reducing sperm quality by increasing oxidative stress and impairing DNA repair mechanism (13). These findings highlight the intricate interplay between lifestyle, environmental factors, and male reproductive health.

In spite of growing global awareness, data on male infertility in Ghana remains sparse and not well established (2). Evidence suggests that long-term exposure to environmental pollutants, combined with dietary and lifestyle modifications, significantly contributes to reduced sperm quality and male infertility (14, 15). Modifiable lifestyle behaviors, such as smoking, alcohol abuse, elevated BMI, poor diet, and physical inactivity, have been implicated in reduced semen quality (16, 17).

The dysregulation of reproductive hormones, including follicle-stimulating hormone (FSH), luteinizing hormone (LH), and testosterone, contributes to the pathogenesis of male infertility (18). Gonadotropin-releasing hormone (GnRH) signals the release of LH and FSH, which regulate testicular function (19). Testosterone aids spermatogenesis and modulates sperm motility, which estrogen acts as a negative feedback regulator of LH and FSH (20). Hormonal anomalies impair spermatogenesis and may be associated with reduced sperm morphology, motility, and concentration (21).

In sub-Saharan Africa, particularly in Ghana, modifiable lifestyle factors affecting male infertility remains under-researched (6). Understanding these factors is essential for developing preventive strategies and improving reproductive outcomes (22). This study investigates the relationship between modifiable lifestyle factors and male reproductive health among attendees of an IVF clinic in Ghana.

## Materials and methods

### Study design and participants

This cross-sectional study was conducted at the Family Health Hospital, Teshie, Ghana. The study recruited 212 male participants who attended the hospital's fertility clinic between November 2022 and April 2023. Ethical approval was obtained from the Ethical and Protocol Review Committee of the University of Ghana (approval number: CHS-Est/M.4-P.5.3/2022–2023). Inclusion criteria included males aged 25–55 years attending the IVF clinic, willing to provide semen and blood samples. Exclusion criteria included known genetic disorders or chronic illnesses affecting fertility, recent febrile illnesses (within the last 3 months), history of vasectomy or other infertility surgeries, use of medications known to impair fertility such as anabolic steroids, and significant lifestyle factors such as heavy substance abuse.

### Data collection

Data were collected using a structured questionnaire that included socio-demographic characteristics, lifestyle factors, and reproductive history. The Hospital Anxiety Depression Scale (HADS) assessed psychological stress anxiety and depression (23). Participant anonymity was maintained through de-identification of data during data collection and analysis.

### Data storage

All collected data were anonymized and stored on password-protected systems. Access was restricted to authorized personnel only. Physical forms such as consent forms were stored in locked cabinets in compliance with institutional ethical standards. Access to sensitive information was restricted to authorized personnel.

#### Questionnaire design

A structured questionnaire that included socio-demographic characteristics, lifestyle factors, and reproductive history. The questionnaire was paper-based, divided into five sections:

#### Demographics

Age, marital status, and education level.

#### Lifestyle factors

Smoking habits, alcohol and caffeine consumption, use of alcoholic bitters and physical activity.

#### Psychological stress

Assessed using the Hospital Anxiety and Depression Scale (HADS).

#### Environmental exposures

Occupational heat exposure, mobile phone use (proximity to reproductive organs), and laptop usage. Data on heat exposure, mobile phone, and laptop use were collected but could not be quantified reliably. As such, these factors were excluded from statistical analysis.

#### Reproductive history

Previous fertility treatments and outcomes.

The questionnaire was adapted from validated instruments, including the Austin Fertility and Reproductive Medicine Center (2015) and Johns Hopkins Bayview Medical Center (2011).

#### Supplementary document

For transparency and replicability, the study questionnaire has been made available as a supplementary document upon request.

### Anthropometric assessment

Body mass index (BMI) was determined by measuring body weight (in kilograms) using a digital weighing scale (Kinlee, China), and height (meters) was measured using a Shanti Stadiometer (Rohit Enterprise, India). The BMI was calculated as weight (kg) divided by height squared (m^2^). Participants were classified according to WHO BMI categories: underweight (BMI < 18.5), normal weight (BMI 18.5–24.9), overweight (BMI 25.0–29.9), and obese (BMI ≥ 30.0).

### Lifestyle assessment questionnaire design

The questionnaire was adapted from validated instruments, including the Austin Fertility and Reproductive Medicine Center (2015) and Johns Hopkins Bayview Medical Center (2011). It comprised sections on participants’ age, smoking habits, alcohol and caffeine consumption, use of alcoholic bitters, occupational heat exposure, and mobile phone usage (including proximity to the reproductive organs) and psychological stress.

### Psychological stress assessment

Psychological stress was evaluated using the “Hospital Anxiety and Depression Scale (HADS)”. HADS is a validated tool comprising 14 items, with seven items assessing anxiety and seven assessing depression. Each item is scored on a 4-point scale (0–3), with total scores ranging from 0 to 21 for both anxiety and depression subscales. The overall HADS score was categorized as follows: 0–7 (non-cases), 8–10 (borderline cases), and 11–21 (clinical cases).

### Semen analysis

Semen samples were analyzed following the WHO 2010 guidelines (24) performed by trained personnel at the Family Health Hospital laboratory, with additional parameters assessed for age-specific trends.

### Patient instructions for semen analysis

The participants were instructed to maintain an abstinence period of (2–5) days before sample collection, following WHO guidelines.

An additional preparation set of instruction such as avoiding caffeine, smoking, or alcohol before sample collection.

### Specific protocols for semen analysis

#### Progressive motility grading

##### Protocol

Semen samples were analyzed using light microscopy at 400× magnification under a phase-contrast microscope pre-warmed to 37°C.

A calibrated slide with a Neubauer counting chamber was used to assess motility.

Progressive motility was graded based on the WHO criteria:

  Grade A: Rapid and progressive forward movement.

  Grade B: Slower or less linear progressive movement.

  Grade C: Non-progressive movement.

  Grade D: Immotile sperm.

#### Sperm viability assessment

##### Protocol

The eosin-nigrosin staining method was used.

A small aliquot of semen was mixed with an equal volume of eosin-nigrosin stain and smeared on a glass slide.

The slide was air-dried and examined under light microscopy at 400× magnification.

Viable sperm remained unstained, while non-viable sperm absorbed the eosin dye.

At least 200 sperm were counted per sample to calculate the percentage viability.

#### Sperm morphology assessment

### Protocol

Morphology was evaluated using Papanicolaou staining (or Diff-Quik staining as an alternative).

Smears of semen samples were prepared, fixed, and stained according to standardized protocols.

Sperm morphology was assessed under light microscopy at 1,000× magnification using oil immersion.

Kruger's strict criteria were applied to classify sperm as normal or abnormal based on head shape, mid-piece, and tail structure.

#### Personnel and inter-observer variability

##### Semen analysis personnel

All analyses were conducted by trained laboratory technologists with expertise in andrology.

Personnel underwent calibration training to ensure proficiency in manual and automated semen analysis methods.

##### Control of inter-observer variability

A standardized protocol was followed to minimize variability, and all staff were blinded to participant identifiers during analysis.

Randomized duplicate samples were re-analyzed by a second technologist, and discrepancies greater than 5% were resolved by a senior analyst.

### Hormonal assay

Fasting blood samples for hormonal assays were collected in the morning between 7:00 and 9:00 A.M. and analyzed using the Mini Vidas multi-parametric immunoassay analyzer (Biomerieux Diagnostics, France). This system, based on the enzyme-linked fluorescent assay (ELFA) method, was selected for its cost-effectiveness and accessibility in low-resource settings as a reliable alternative to mass spectrometry, the gold standard for hormonal analysis (25). Validation studies, including Hammoud et al. (2008), have demonstrated the Mini-Vidas system's sensitivity (>85%) and specificity (87%) compared to mass spectrometry. To ensure robustness, quality controls and calibration protocols were employed during the hormonal assays. While ELFA provides reliable results for clinical research, its lower accuracy in detecting subtle hormonal variations could influence findings related to borderline imbalances. However, within the context of the study, ELFA's reliability is sufficient to meet its objectives.

### Sample size rationale and confounders

The sample size of 212 was determined based on prior studies, assessing semen quality in similar populations. The 212 participants represent approximately 60% of all males attending the clinic during the study period, selected through convenience sampling. This approach ensures representation of common clinic demographics but may introduce selection bias. A 10% margin was added to account for potential dropouts. Confounding variables, such as age and pre-existing medical conditions (genetic disorders, chronic illnesses and diseases known to affect fertility) were accounted for in the analysis.

### Statistical analysis

Data were analyzed using SPSS (v26.0). Confidence intervals 95% were calculated for all findings. Descriptive statistics summarized baseline characteristics. Correlations between lifestyle factors, HADS scores, and semen parameters were analyzed using multivariate regression models. Pearson's product-moment correlation was employed to assess relationships between lifestyle factors, semen parameters, and hormonal levels. To control for multiple comparisons, the Bonferroni correction was applied, with a family-wise error rate set at *α* = 0.05 (26). While Bonferroni correction effectively controls for Type I error, its conservatism risks Type II errors, particularly in exploratory analyses. Future studies could benefit from using FDR to balance sensitivity and specificity. Future analyses could employ regression models to adjust for potential confounders such as age and BMI, providing deeper insights into these relationships.

## Results

**Figure 1:**
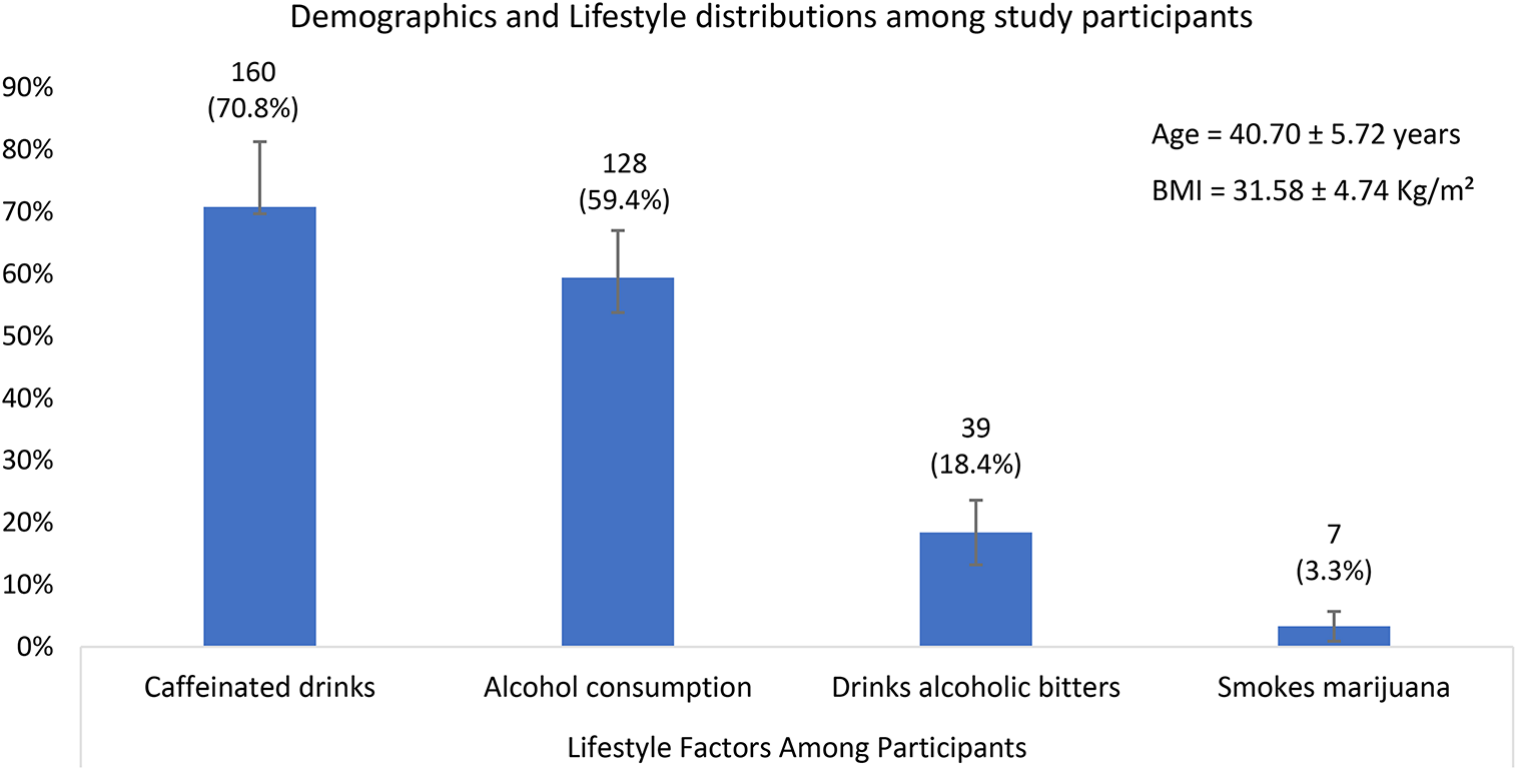
A bar graph showing the descriptive statistics of Demographics and Lifestyle distributions among study participants. With 95% confidence intervals.

A total of 212 male participants attending the Family Health Hospital fertility center in Teshie, Ghana, were included in the study. The mean age of participants was (40.7 ± 5.7) years, and the mean BMI was (31.6 ± 4.7) kg/m^2^, indicating that many participants were overweight or obese (Figure 1). Nearly 60% of participants consumed alcohol, and 15% were current smokers. About 18.4% reported consumption of alcoholic bitters and 70.8% consumed caffeinated drinks while 3.3% smoked marijuana. The psychological stress levels, assessed using the Hospital Anxiety and Depression Scale (HADS), recorded a mean score of 12.74 (range: 5–15) (Figure 1).

### Semen parameters

**Figure 2:**
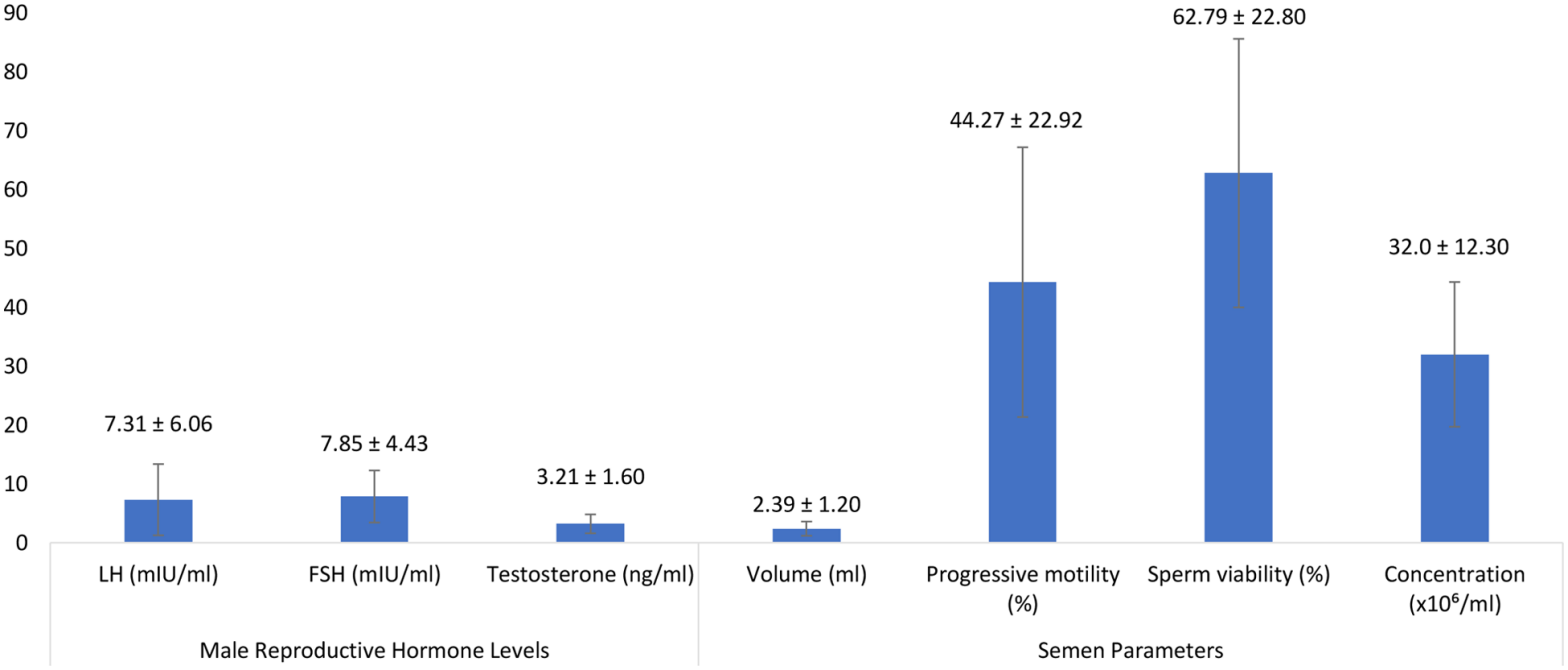
A bar graph showing the descriptive statistics of Male Reproductive Hormone Levels and Semen Parameters Among Study Participants.

Sperm concentration and motility were significantly lower in smokers compared to non-smokers (*p* = 0.010). Caffeine consumption was positively correlated with sperm motility (*r* = 0.399, *p* < 0.01) (Figure 2). While alcohol consumption showed a marginal association with reduced sperm concentration (*p* = 0.054), it does not meet the threshold for statistical significance (*p* < 0.05). This finding suggests a trend rather than a definitive conclusion, warranting further investigation with larger sample sizes.

### Hormonal levels

Mean testosterone concentration was (3.21 ± 1.60) ng/ml, with luteinizing hormone (LH) and follicle-stimulating hormone (FSH) levels averaging (7.31 ± 6.06) mIU/ml and (7.85 ± 4.43) mIU/ml, respectively. Elevated BMI was inversely associated with testosterone levels (*r* = −0.424, *p* < 0.05).

### Lifestyle factors and semen parameters

Among the participants, 3.3% smoked marijuana. However, its impact on semen parameters, such as sperm motility, viability, or concentration, remains unexplored in the data. Smoking and psychological stress were significantly associated with reduced sperm motility, viability, and concentration (*p* < 0.05) (27). Elevated BMI negatively correlated with sperm concentration and testosterone levels (*p* < 0.05), while caffeine consumption positively influenced sperm motility. Elevated BMI negatively correlated with sperm morphology and concentration, while testosterone levels were inversely associated with laptop use on laps (*p* < 0.01) (28).

Vigorous physical exercise showed mixed effects, with moderate activity correlating with improved semen quality.

A negative correlation was observed between age and progressive sperm motility (*r* = −0.379, *p* < 0.01) and sperm morphology (*r* = −0.231, *p* = 0.001). However, a positive association was found between age and sperm non-progressive motility (*r* = 0.367, *p* = 0.000) and immotility (*r* = 0.225, *p* = 0.001).

The BMI also demonstrated significant correlations with semen parameters. A negative correlation was observed between BMI and sperm motility (progressive: *r* = −0.274, *p* = 0.000; non-progressive: *r* = −0.185, *p* = 0.009) as well as morphology (*r* = −0.403, *p* = 0.004). Conversely, BMI positively correlated with semen volume (*r* = 0.216, *p* = 0.002) and sperm viability (*r* = 0.207, *p* = 0.003).

Alcohol consumption was weakly negatively correlated with sperm concentration but positively associated with semen volume (*r* = 0.313, *p* = 0.000). Alcoholic bitters consumption significantly reduced progressive motility (*r* = −0.685, *p* = 0.000) and sperm morphology (*r* = −0.585, *p* = 0.000). Caffeine intake showed a moderate positive correlation with sperm viability (*r* = 0.207, *p* = 0.003) and sperm concentration (*r* = 0.223, *p* = 0.001) but negatively correlated with non-progressive motility (*r* = −0.437, *p* = 0.000) and immotile sperm (*r* = −0.219, *p* = 0.002) ((Table 1).

Cigarette smoking had a moderate positive association with immotile sperm (*r* = 0.451, *p* = 0.000) and a negative association with progressive motility (*r* = −0.317, *p* = 0.000) and sperm viability (*r* = −0.450, *p* = 0.000). Psychological stress, as assessed by HADS, negatively correlated with progressive motility (*r* = −0.269, *p* = 0.000) and sperm concentration (*r* = −0.273, *p* = 0.001), while positively correlating with immotile sperm (*r* = 0.285, *p* = 0.000).

### Correlations with hormonal levels

A significant positive correlation was observed between age and FSH (*r* = 0.365, *p* = 0.000) and estradiol (*r* = 0.361, *p* = 0.000) (Table 2) BMI was positively correlated with LH (*r* = 0.044, *p* = 0.527), testosterone (*r* = 0.365, *p* = 0.000), and estradiol (*r* = 0.361, *p* = 0.000). Alcohol consumption exhibited a positive association with FSH (*r* = 0.143, *p* = 0.038) and estradiol (*r* = 0.159, *p* = 0.021), while caffeine consumption showed strong negative correlations with LH, FSH, and estradiol (*p* < 0.001). Cigarette smoking significantly correlated with FSH (*p* < 0.05), while laptop use on the lap showed a negative correlation with testosterone and LH (*p* < 0.05).

**Table 2:** Correlation between lifestyle, environmental factors, and reproductive hormones.

Lifestyle factors	LH	FSH	Testosterone	Estradiol
Age of starting fatherhood	−0.044	0.365^a^	−0.045	0.361^a^
Body mass index	0.266^a^	0.078	0.338^a^	0.271^a^
Alcohol	0.013	0.143^b^	0.025	0.159^b^
Alcoholic bitters	0.026	0.162^b^	0.427^a^	−0.012
Caffeinated drinks	−0.557^a^	−0.443^a^	−0.019	−0.209^a^
Cigarette smoking	0.001	0.246^a^	−0.072	0.099
Psychological stress (HADS)	0.289^a^	0.349^a^	0.284^a^	0.280^a^

^a^ Correlation is significant at the *P* < 0.01 level (2-tailed). ^b^Correlation is significant at the *P* < 0.05 level (2-tailed). Cor. Coeff, r, correlation coefficient; LH, luteinizing hormone; FSH, follicle stimulating hormone.

#### HADS and semen parameters

The HADS scores highlight moderate levels of anxiety and depression, negatively correlating with sperm quality. Table 3 shows the correlation between HADS scores and semen parameters. Higher anxiety and depression scores were associated with reduced sperm motility and concentration (*p* < 0.05). Participants aged 40 years and above exhibited significantly higher HADS scores, correlating with poorer semen quality.

**Table 3:** HADS and semen parameters.

HADS score	Progressive motility (%)	Sperm viability (%)	Sperm concentration (million/Ml)
Low (0–7)	58.3 ± 12.4	76.2 ± 9.3	32.1 ± 8.5
Moderate (8–10)	44.7 ± 14.6	62.8 ± 12.7	24.3 ± 6.1
High (11–21)	36.5 ± 18.2	51.3 ± 15.8	18.7 ± 5.2

## Discussion

The study confirms the significant impact of modifiable lifestyle factors on male reproductive health. Smoking and psychological stress were strongly associated with reduced sperm motility and concentration (2). Elevated BMI negatively correlated with sperm morphology consistent with global trends linking obesity to male infertility (29).

The inverse relationship between BMI and sperm concentration is consistent with evidence suggesting adiposity-induced testosterone reduction and increased estradiol levels, which disrupt spermatogenesis (30). Addressing obesity through lifestyle interventions could significantly enhance fertility outcomes. This makes it imperative for urgent need for public health initiatives to address obesity and its effects on reproductive health. Obesity significantly modulates genetic and epigenetic factors, disrupting sperm morphology and function. Recent studies show that obesity-induced hormonal imbalances alter testosterone and estradiol levels, impairing spermatogenesis (31). Adipose tissue acts as an endocrine organ, increasing aromatase activity and converting testosterone into estradiol, thus reducing androgen levels necessary for sperm production. Chronic inflammation associated with obesity elevates pro-inflammatory cytokines like TNF-alpha and IL-6, exacerbating oxidative stress and DNA damage in sperm cells. Elevated scrotal temperatures from excessive adiposity further impair spermatogenesis and sperm motility. Weight reduction through lifestyle interventions, including dietary changes and moderate physical activity, has been shown to improve hormonal profiles and semen quality. These findings underscore the need for incorporating weight management into fertility treatment protocols.

Caffeine's role in oxidative stress is well-documented. Excessive consumption increases reactive oxygen species (ROS), leading to sperm DNA fragmentation (32). Recent studies suggest caffeine consumption exhibits a dose-dependent effect on sperm quality. Moderate intake has been associated with improved motility, potentially due to enhanced mitochondrial function. However, excessive caffeine intake may lead to increased reactive oxygen species (ROS), inducing oxidative stress and DNA fragmentation. Ricci et al. reported risks of chromosomal abnormalities and pre-zygotic DNA damage linked to caffeine overconsumption (33). These findings document the need for individualized recommendations on caffeine intake in fertility treatments. Similarly, smokers also showed elevated BPDE-DNA adducts, which impair DNA repair pathway, further exacerbating infertility risk.

Lifestyle interventions, including moderate exercise, stress management, and dietary antioxidants, is key to improving semen quality. Personalized care approaches tailored to individual risk factors, such as antioxidant therapy and hormonal modulation, are critical for optimizing reproductive health outcomes.

### Socio-demographic, anthropometric characteristics, and lifestyle of participants

All participants were married or in committed relationships, with a mean age of (40.7 ± 5.7) years, suggesting that they sought medical help for infertility at an advanced age. Table 4 shows specific age group and semen parameters in that, Increased age showed decreased semen parameters. This supports findings by Sharma et al., which showed that male fertility declines with age, emphasizing the importance of considering age in family planning (15). The average BMI indicated obesity among participants, possibly due to the economic status associated with affording expensive treatments such as *in vitro* fertilization (IVF) (27).

**Table 4:** Age-specific semen parameters.

Age group	Progressive motility (%)	Sperm viability (%)	Morphology (%)	Concentration (x10⁶/ml)
25–34 years	58.3 ± 12.4	76.2 ± 9.3	15.3 ± 5.1	32.1 ± 8.5
35–44 years	44.7 ± 14.6	62.8 ± 12.7	13.2 ± 4.8	24.3 ± 6.1
45–54 years	36.5 ± 18.2	51.3 ± 15.8	11.5 ± 4.2	18.7 ± 5.2

More than half of the participants consumed alcohol and caffeinated beverages. Additionally, the hospital anxiety and depression scale (HADS) revealed that participants were already stressed, possibly due to the pressure of infertility at an older age, social stigma, and low self-esteem (28).

### Relationship between socio-demographics, lifestyle activities, and semen parameters

This study found that advancing paternal age negatively impacts sperm progressive motility, viability, and morphology, while increasing the number of non-progressive and immotile spermatozoa. These results are consistent with Dunson et al., who reported a decline in semen quality characteristics as paternal age exceeds 35 years (34). Pino et al. also reported increased sperm DNA damage and decreased viability in men over 40 years of age (35).

A higher BMI was associated with increased semen volume and immotile spermatozoa, but reduced sperm viability, progressive motility, morphology, and concentration, as similarly observed by Sallmen et al. who found that a three-unit increase in BMI decreased male fertility (36). Alcohol consumption, though not statistically significant for most semen parameters, showed a moderate positive association with semen volume and a weak negative association with sperm concentration. The absence of a substantial impact of alcohol on sperm quality is consistent with findings by Muthusami and Chinnaswamy, which found no significant differences in semen parameters between alcoholics and non-alcoholics (37).

Alcoholic bitters had a detrimental effect on progressive motility, morphology, and viability, but improved semen volume. Caffeinated beverage consumption showed a beneficial relationship with progressive motility, morphology, and concentration but had a negative correlation with non-progressive motility and immotile sperm. These results align with Marshburn et al., who reported improved sperm motility with increased coffee consumption (38). However, Ricci et al. suggested caffeine could reduce male fertility through sperm aneuploidy and DNA damage, though the data remains inconsistent (33).

Cigarette smoking showed a strong negative correlation with sperm progression, viability, and morphology, while having a positive relationship with non-progressive and immotile spermatozoa. These findings is consistent with earlier studies (2, 39) demonstrating smoking's negative impact on semen parameters. While the impact of marijuana on semen parameters was not explored directly in this study, existing evidence indicates that cannabis use adversely affects sperm quality. Studies have shown that moderate to heavy marijuana use is associated with reduced sperm motility, concentration, and abnormal morphology. For instance, Gundersen et al. found that marijuana use more than once per week was linked to a 29% reduction in sperm concentration in a cohort of young Danish men (40). Similarly, a study by Barazani et al. concluded that tetrahydrocannabinol (THC), the primary psychoactive compound in cannabis, negatively affects the hypothalamic-pituitary-gonadal axis, impairing spermatogenesis and resulting in decreased sperm count and motility (41). Furthermore, Kolodny et al. provided early evidence that chronic cannabis use is associated with abnormalities in sperm morphology and overall male fertility (42).

Psychological stress showed a significant negative correlation with sperm progressive motility, morphology, viability, and concentration. Bhongade et al. also found that stress decreases testosterone levels, leading to reduced sperm count, motility, and normal morphology (31).

### Relationship between socio-demographics, lifestyle activities, and reproductive hormones

Age was positively correlated with FSH and estradiol, but showed no significant relationship with LH and testosterone, unlike previous report by Gunes et al., observed declines in testicular function and testosterone levels in aging men (43). The BMI positively correlated with LH, testosterone, and estradiol, but showed no significant relationship with FSH. MacDonald et al. similarly reported a relationship between BMI and testosterone (44), and Hakonsen et al. found increased BMI was associated with decreased testosterone and increased estradiol (45).

Alcohol consumption showed a positive relationship with FSH and testosterone, but no significant association with LH and estrogen. This contradicts earlier studies that linked alcohol to decreased LH and FSH (46), though it could be due to the moderate alcohol consumption in this cohort. Alcoholic bitters consumption was positively associated with testosterone but had a weak relationship with LH, FSH, and estrogen.

Caffeine consumption negatively correlated with LH, FSH, and estrogen but had no significant impact on testosterone. Oluwole et al. found similar results in rats, where caffeine decreased LH and FSH but did not significantly alter testosterone (47). The inhibitory effect of caffeine on the aromatase enzyme may explain the unchanged testosterone levels.

### Relationship between modifiable lifestyles and reproductive hormones

Psychological stress positively correlated with LH, FSH, testosterone, and estrogen, possibly due to stress-induced hormone release through the hypothalamic-pituitary-adrenal axis. Bhongade et al. similarly found elevated FSH and LH in men under psychological stress, though stress also leads to Leydig cell apoptosis and reduced testosterone levels (31).

In summary, the study highlights the significant impact of lifestyle factors on male reproductive health. Psychological stress, environmental pollutants and endocrine-disrupting chemicals emerged as key contributors to declining semen quality. Sperm DNA damage, exacerbated by smoking and caffeine, has profound implications for ART outcomes.

Recent evidence supports personalized care approaches, incorporating lifestyle modifications, psychological support, and antioxidant therapies. These therapies, such as Coenzyme Q10, selenium, and zinc supplementation, have shown efficacy in reducing oxidative stress and improving sperm parameters. Majzoub & Agarwal (2018) demonstrated significant improvements in motility and DNA integrity with these interventions (48). Personalized care models integrating stress management, dietary adjustments, and targeted antioxidant therapies offer promising avenues for enhancing fertility outcomes.These interventions can enhance semen parameters and improve ART success rates.

## Conclusions

This study highlights the significant impact of modifiable lifestyle factors, including smoking, psychological stress, and elevated BMI, on male reproductive health. Public health initiatives should focus on promoting healthier lifestyles and mitigating environmental risks to improve fertility outcomes.

Specifically, increasing age was identified as a poor predictor of sperm quality in fertility management, emphasizing the need for awareness among Ghanaian men regarding the importance of fatherhood prior to the age of 40 years. In contrast, alcohol consumption did not show a significant association with sperm quality, while caffeine intake was linked to improved progressive motility, morphology, and sperm concentration.

Elevated psychological stress was associated with changes in hormone levels, some of which negatively affected semen quality.

These findings highlight the critical impact of modifiable lifestyle factors such as smoking, psychological stress, and elevated BMI on male reproductive health. To address this issue:

• Governments and policymakers should prioritize public health initiatives that promote healthy lifestyle behaviors, reduce environmental risks, and improve mental health support.
• Reproductive health education should be integrated into sustainable development goals (SDGs) to mitigate the long-term societal impact of declining male fertility.
• Personalized care strategies focusing on antioxidant therapies and stress management should be incorporated into clinical practice to optimize fertility outcomes.

### Strengths and limitations

#### Strengths

• First comprehensive analysis of lifestyle factors and reproductive health in Ghana.

#### Limitations

• Cross-sectional design limits causal inferences.
• Small sample size may affect generalizability.
• ELFA-based hormonal assays may lack the precision of mass spectrometry.
• This study was unable to include variables such as heat exposure, mobile phone, and laptop use due to the lack of quantitative metrics, limiting the comparability and statistical analysis of their impact on semen parameters.

#### Implications for public health

Findings from this study highlight the importance of addressing modifiable lifestyle factors to improve male reproductive health. Considering the increased incidence of male infertility and the high costs associated with assisted reproductive technologies, public health interventions should focus on promoting healthier lifestyles and reducing exposure to environmental risks. Education on the detrimental effects of smoking, excessive alcohol consumption, and stress management could significantly enhance fertility outcomes. Furthermore, the role of electronic devices (lab top and mobile phones) in male infertility requires further investigation to clarify conflicting findings.

#### Future research directions

Further longitudinal studies with detailed exposure metrics to provide more robust insights into the impact of lifestyle factors on male reproductive healthcare needed to explore the long-term effects of lifestyle and environmental exposures on male fertility, particularly in under-researched regions like sub-Saharan Africa. Future research should investigate the molecular mechanisms underlying BMI's impact on testosterone levels and sperm concentration, using interventions like antioxidant therapies and hormonal modulation.

## Data Availability

The raw data supporting the conclusions of this article will be made available by the authors, without undue reservation.

## References

[1] World Health Organization. WHO Fact Sheet on Infertility. Philadelphia, PA: LWW (2021). p. e52.

[2] Blaha M Nemcova L Prochazka R. Cyclic guanosine monophosphate does not inhibit gonadotropin-induced activation of mitogen-activated protein kinase 3/1 in pig cumulus-oocyte complexes. Reprod Biol Endocrinol. (2015) 13:1–12. 10.1186/1477-7827-13-1 25567742 PMC4293816

[3] Mascarenhas MN Flaxman SR Boerma T Vanderpoel S Stevens GA. National, regional, and global trends in infertility prevalence since 1990: a systematic analysis of 277 health surveys. PLoS Med. (2012) 9(12):e1001356. 10.1371/journal.pmed.1001356 23271957 PMC3525527

[4] Levine H Jørgensen N Martino-Andrade A Mendiola J Weksler-Derri D Mindlis I Temporal trends in sperm count: a systematic review and meta-regression analysis. Hum Reprod Update. (2017) 23(6):646–59. 10.1093/humupd/dmx022 28981654 PMC6455044

[5] Uadia PO Emokpae AM. Male infertility in Nigeria: a neglected reproductive health issue requiring attention. J Basic Clin Reprod Sci. (2015) 4(2):45–53. 10.4103/2278-960X.161042

[6] Osei NY. Need for accessible infertility care in Ghana: the patients’ voice. Facts Views Vis Obgyn. (2016) 8(2):125. 27909570 PMC5130302

[7] Nordkap L Joensen UN Jensen MB Jørgensen N. Regional differences and temporal trends in male reproductive health disorders: semen quality may be a sensitive marker of environmental exposures. Mol Cell Endocrinol. (2012) 355(2):221–30. 10.1016/j.mce.2011.05.048 22138051

[8] Bornman R Aneck-Hahn N. Endocrine disruptors and male infertility. In: du Plessis SS Agarwal A Sabanegh ES Jr., editors. Male Infertility: A Complete Guide to Lifestyle and Environmental Factors. New York, NY: Springer New York (2014). p. 193–210.

[9] Meeker JD Godfrey-Bailey L Hauser R. Relationships between serum hormone levels and semen quality among men from an infertility clinic. J Androl. (2007) 28(3):397–406. 10.2164/jandrol.106.001545 17135633

[10] Le Magueresse-Battistoni B Labaronne E Vidal H Naville D. Endocrine disrupting chemicals in mixture and obesity, diabetes and related metabolic disorders. World J Biol Chem. (2017) 8(2):108. 10.4331/wjbc.v8.i2.108 28588754 PMC5439162

[11] Gebhart MB Hines RS Penman A Holland AC. How do patient perceived determinants influence the decision-making process to accept or decline preimplantation genetic screening? Fertil Steril. (2016) 105(1):188–93. 10.1016/j.fertnstert.2015.09.022 26474735

[12] Ibanez-Perez J Santos-Zorrozua B Lopez-Lopez E Matorras R Garcia-Orad A. An update on the implication of physical activity on semen quality: a systematic review and meta-analysis. Arch Gynecol Obstet. (2019) 299:901–21. 10.1007/s00404-019-05045-8 30671700

[13] Hadwan MH Almashhedy LA Alsalman ARS. Study of the effects of oral zinc supplementation on peroxynitrite levels, arginase activity and NO synthase activity in seminal plasma of Iraqi asthenospermic patients. Reprod Biol Endocrinol. (2014) 12:1–8. 10.1186/1477-7827-12-1 24383664 PMC3882288

[14] Mima M Greenwald D Ohlander S. Environmental toxins and male fertility. Curr Urol Rep. (2018) 19:1–8. 10.1007/s11934-018-0804-1 29774504

[15] Porcu G Lehert P Colella C Giorgetti C. Predicting live birth chances for women with multiple consecutive failing IVF cycles: a simple and accurate prediction for routine medical practice. Reprod Biol Endocrinol. (2013) 11:1–6. 10.1186/1477-7827-11-1 23302328 PMC3551786

[16] Ramaraju GA Teppala S Prathigudupu K Kalagara M Thota S Kota M Association between obesity and sperm quality. Andrologia. (2018) 50(3):e12888. 10.1111/and.12888 28929508

[17] Salas-Huetos A Bulló M Salas-Salvadó J. Dietary patterns, foods and nutrients in male fertility parameters and fecundability: a systematic review of observational studies. Hum Reprod Update. (2017) 23(4):371–89. 10.1093/humupd/dmx006 28333357

[18] Krausz C Rosta V Swerdloff RS Wang C. Genetics of male infertility. In: Pyeritz RE Korf BR Grody WW, editors. Emery and Rimoin’s Principles and Practice of Medical Genetics and Genomics. Cambridge, MA: Academic Press (2022). p. 121–47. 10.1016/C2017-0-01777-9

[19] Melmed S Polonsky KS Larsen PR Kronenberg HM. Williams Textbook of Endocrinology E-Book. Amsterdam: Elsevier Health Sciences (2015).

[20] Walker WH. Testosterone signaling and the regulation of spermatogenesis. Spermatogenesis. (2011) 1(2):116–20. 10.4161/spmg.1.2.16956 22319659 PMC3271653

[21] Esteves SC Miyaoka R Agarwal A. An update on the clinical assessment of the infertile male. Clinics. (2011) 66(4):691–700. 10.1590/S1807-59322011000400026 21655766 PMC3093801

[22] Bocu K Boeri L Mahmutoglu AM Vogiatzi P. Can lifestyle changes significantly improve male fertility: a narrative review? Arab J Urol. (2024):1–11. 10.1080/20905998.2024.2421626 PMC12308861 40747470

[23] Michopoulos I Douzenis A Kalkavoura C Christodoulou C Michalopoulou P Kalemi G Hospital anxiety and depression scale (HADS): validation in a Greek general hospital sample. Ann Gen Psychiatry. (2008) 7:1–5. 10.1186/1744-859X-7-4 18325093 PMC2276214

[24] World Health Organization. WHO Laboratory Manual for the Examination and Processing of Human Semen. Geneva: World Health Organization (2021).

[25] Dey MK Iftesum M Devireddy R Gartia MR. New technologies and reagents in lateral flow assay (LFA) designs for enhancing accuracy and sensitivity. Anal Methods. (2023) 15(35):4351–76. 10.1039/D3AY00844D 37615701

[26] Field A. Discovering Statistics Using IBM SPSS Statistics. London: Sage (2013).

[27] Service CA Puri D Al Azzawi S Hsieh TC Patel DP. The impact of obesity and metabolic health on male fertility: a systematic review. Fertil Steril. (2023) 120(6):1098–111. 10.1016/j.fertnstert.2023.10.017 37839720

[28] Tavousi SA Behjati M Milajerdi A Mohammadi AH. Psychological assessment in infertility: a systematic review and meta-analysis. Front Psychol. (2022) 13:961722. 10.3389/fpsyg.2022.961722 36389481 PMC9650266

[29] Bieniek JM Kashanian JA Deibert CM Grober ED Lo KC Brannigan RE Influence of increasing body mass index on semen and reproductive hormonal parameters in a multi-institutional cohort of subfertile men. Fertil Steril. (2016) 106(5):1070–5. 10.1016/j.fertnstert.2016.06.041 27460460

[30] Bellastella G Menafra D Puliani G Colao A Savastano S, Obesity Programs of nutrition, Education, Research and Assessment (OPERA) Group. How much does obesity affect the male reproductive function? Int J Obes Suppl. (2019) 9(1):50–64. 10.1038/s41367-019-0008-2 31391924 PMC6683183

[31] Bhongade MB Prasad S Jiloha RC Ray PC Mohapatra S Koner BC. Effect of psychological stress on fertility hormones and seminal quality in male partners of infertile couples. Andrologia. (2015) 47(3):336–42. 10.1111/and.12268 24673246

[32] Wright C Milne S Leeson H. Sperm DNA damage caused by oxidative stress: modifiable clinical, lifestyle and nutritional factors in male infertility. Reprod Biomed Online. (2014) 28(6):684–703. 10.1016/j.rbmo.2014.02.004 24745838

[33] Ricci E Viganò P Cipriani S Somigliana E Chiaffarino F Bulfoni A Coffee and caffeine intake and male infertility: a systematic review. Nutr J. (2017) 16:1–14. 10.1186/s12937-017-0257-2 28646871 PMC5482951

[34] Dunson DB Baird DD Colombo B. Increased infertility with age in men and women. Obstet Gynecol. (2004) 103(1):51–6. 10.1097/01.AOG.0000100153.24061.45 14704244

[35] Pino V Sanz A Valdés N Crosby J Mackenna A. The effects of aging on semen parameters and sperm DNA fragmentation. JBRA Assist Reprod. (2020) 24(1):82. 10.5935/1518-0557.20190058 31692316 PMC6993171

[36] Sallmén M Sandler DP Hoppin JA Blair A Baird DD. Reduced fertility among overweight and obese men. Epidemiology. (2006) 17(5):520–3. 10.1097/01.ede.0000229953.76862.e5 16837825

[37] Muthusami KR Chinnaswamy P. Effect of chronic alcoholism on male fertility hormones and semen quality. Fertil Steril. (2005) 84(4):919–24. 10.1016/j.fertnstert.2005.04.025 16213844

[38] Marshburn PB Sloan CS Hammond MG. Semen quality and association with coffee drinking, cigarette smoking, and ethanol consumption. Fertil Steril. (1989) 52(1):162–5. 10.1016/S0015-0282(16)60809-9 2744185

[39] Harlev A Agarwal A Gunes SO Shetty A du Plessis SS. Smoking and male infertility: an evidence-based review. World J Mens Health. (2015) 33(3):143–60. 10.5534/wjmh.2015.33.3.143 26770934 PMC4709430

[40] Gundersen TD Jørgensen N Andersson AM Bang AK Nordkap L Skakkebæk NE Association between use of marijuana and male reproductive hormones and semen quality: a study among 1,215 healthy young men. Am J Epidemiol. (2015) 182(6):473–81. 10.1093/aje/kwv135 26283092

[41] Barazani Y Katz BF Nagler HM Stember DS. Lifestyle, environment, and male reproductive health. Urol Clin. (2014) 41(1):55–66. 10.1016/j.ucl.2013.08.017 24286767

[42] Kolodny RC Masters WH Kolodner RM Toro G. Depression of plasma testosterone levels after chronic intensive marihuana use. N Engl J Med. (1974) 290(16):872–4. 10.1056/NEJM197404182901602 4816961

[43] Gunes S Hekim GNT Arslan MA Asci R. Effects of aging on the male reproductive system. J Assist Reprod Genet. (2016) 33:441–54. 10.1007/s10815-016-0663-y 26867640 PMC4818633

[44] Macdonald AA Stewart AW Farquhar CM. Body mass index in relation to semen quality and reproductive hormones in New Zealand men: a cross-sectional study in fertility clinics. Hum Reprod. (2013) 28(12):3178–87. 10.1093/humrep/det379 24129611

[45] Håkonsen LB Thulstrup AM Aggerholm AS Olsen J Bonde JP Andersen CY Does weight loss improve semen quality and reproductive hormones? Results from a cohort of severely obese men. Reprod Health. (2011) 8:1–8. 10.1186/1742-4755-8-24 21849026 PMC3177768

[46] Moosazadeh M Heydari K Rasouli K Azari S Afshari M Barzegari S Association of the effect of alcohol consumption on luteinizing hormone (LH), follicle-stimulating hormone (FSH), and testosterone hormones in men: a systematic review and meta-analysis. Int J Prev Med. (2024) 15:75. 10.4103/ijpvm.ijpvm_81_24 39867254 PMC11759224

[47] Oluwole OF Salami SA Ogunwole E Raji Y. Implication of caffeine consumption and recovery on the reproductive functions of adult male wistar rats. J Basic Clin Physiol Pharmacol. (2016) 27(5):483–91. 10.1515/jbcpp-2015-0134 27159917

[48] Majzoub A Agarwal A. Systematic review of antioxidant types and doses in male infertility: benefits on semen parameters, advanced sperm function, assisted reproduction and live-birth rate. Arab J Urol. (2018) 16(1):113–24. 10.1016/j.aju.2017.11.013 29713542 PMC5922223

